# BMI is associated with sperm quality and sex hormones in men: a meta-analysis

**DOI:** 10.3389/fendo.2025.1714019

**Published:** 2025-12-05

**Authors:** Fengming Ji, Bing Yan, Chenghao Zhanghuang, Chengchuang Wu, Jinshuan Dai, Shengde Wu

**Affiliations:** 1 Urology Surgery Department Children’s Hospital of Chongqing Medical University, National Clinical Research Center for Child Health and Disorders, Ministry of Education Key Laboratory of Child Development and Disorders, Chongqing Key Laboratory of Structural Birth Defect and Reconstruction, Chongqing, China; 2 Urology Surgery Department of the Children’s Hospital of Kunminng Medical University, Kunming Chlidren’s Hospital, Key Laboratory of Children’s Major Disease Research of Yunnan Province, Kunming, Yunnan, China

**Keywords:** BMI, sperm quality, sex hormones, men, meta-analysis

## Abstract

**Aim:**

To systematically evaluate the relationship between body mass index (BMI) and sperm quality parameters as well as sex hormones levels in males.

**Method:**

A comprehensive literature search was conducted across Pubmed, Embase and the Cochrane Library for literature, China National Knowledge Infrastructure, and Wanfang database. Studies investigating the association between BMI and semen parameters or sex hormones in adult males (>18 years) were included. Two reviewers independently performed study selection, data extraction, and quality evaluation Meta-analysis was performed using RevMan 5.4 and Stata 18.0.

**Results:**

Of the 275 studies identified, 14 met the inclusion criteria. A total of 14 studies involving 8443 patients were included, including 3467 cases of normal BMI, 3444 cases of overweight and 1532 cases were obesity. All 14 studies involved sperm quality analysis, and 4 studies addressed sex hormone analysis. The meta-analysis results indicate that there were statistically significant differences in normal morphology (NM), total motility (TM), sperm concentration (SC), progressive motility (PM), volume and total sperm count (TSC) among the three groups. In the analysis of sex hormones, total testosterone (TT), follicle stimulating hormone (FSH) and luteinizing hormone (LH) showed statistically significant difference among three groups.

**Conclusion:**

Elevated BMI is significantly associated with impaired sperm quality and altered sex hormone levels. BMI should be considered a risk factor in male fertility assessments. Further longitudinal studies are needed to explore the reversibility of these effects through lifestyle interventions.

## 1 Introduction

Infertility is a significant global public-health concern and a primary contributor to demographic unease, and approximately 10%-15% of couples worldwide are impacted. Concurrently, in China, the prevalence among couples of reproductive age has dramatically increased from 2.5%-3% two decades ago to 15%-18% in more recent years (1). Male factors account for nearly half of all infertility cases. Studies report that global total sperm count (TSC) and concentration have declined by 50%- 60% over the past few decades and continue to fall. Data from China show that between 2001 and 2015 the proportion of qualified sperm donors dropped from 56% to 18%, while sperm concentration (SC), progressive motility (PM), and normal morphology (NM) all decreased by approximately 30–60% (2).

The World Health Organization (WHO) has announced that one billion people worldwide are now facing the threat of obesity (3, 4). Evidence shows that, compared with normal weight men, those who are overweight or obese have significantly poorer semen quality, and a higher BMI negatively affects TSC, total motility (TM), NM and testosterone levels (5, 6). While previous investigations have explored the relationship between body mass index (BMI) and semen quality, their results have been incongruent, suggesting that the underlying mechanisms and pathways by which BMI affects these factors are still not fully understood. We therefore conducted a systematic review and meta-analysis to quantify the impact of BMI on semen quality and reproductive hormone levels in men.

## 2 Materials and methods

### 2.1 Search strategy

This systematic review and meta-analysis were conducted in accordance with the Preferred Reporting Items for Systematic Review and Meta-Analysis (PRISMA) guidelines. We searched the databases of Pubmed, Embase, the Cochrane Library, China National Knowledge Infrastructure, and Wanfang database for literature, with the search period covering from 2011 to now. The search terms included: body mass index, BMI, weight, overweight, obesity, obese, sperm, semen, sex hormones.

### 2.2 Inclusion criteria

(1) Study type: Cohort or retrospective studies comparing sperm quality and/or sex hormone levels among men with different BMI status. (2) Study object: adult males (>18 years). (3) Comparator: Comparisons should be made between the different BMI categories (normal weight, overweight, and obesity) regarding sperm quality and hormone levels. Normal weight: 18.5–24.9 kg/m², overweight: 25.0–29.9 kg/m², and obesity: ≥30.0 kg/m² (7). (4) Semen samples were analyzed following the WHO laboratory manual for the examination and processing of human semen.

### 2.3 Exclusion criteria

(1) Non-adult participants (under 18 years of age). (2) Studies that do not differentiate between BMI categories or do not report on the specified BMI classifications. (3) Non-observational studies, including reviews, meta-analyses, case reports, or letters to the editor. (4) Studies lacking quantitative data on sperm quality and hormone levels or without a clear methodology for measuring these outcomes. (5) Studies that do not report at least two indicators of sperm quality or two indicators of hormone levels as specified.

### 2.4 Literature screening, data extraction and quality evaluation

Data were independently extracted by two reviewers and cross-checked, any disagreements were resolved through discussion with a third reviewer. The following information was collected: (1). General study information: first author, year of study, country. (2). General study characteristics: study design, number of participants in each BMI category, age of participants, source of participant recruitment. (3). Outcome measures: volume, SC, PM, NM, TM, TSC and sperm DNA fragmentation (SDF) rate. Sex hormones: total testosterone (TT), estradiol (E2), follicle stimulating hormone (FSH) and luteinizing hormone (LH).

### 2.5 Evaluation of literature quality

Two qualified reviewers independently screened the literature and extracted the data. Any disagreements were resolved by discussion with a third reviewer. The final quality score for each included study was the mean of the two reviewers’ ratings. The widely-used Agency for Healthcare Research and Quality (AHRQ) checklist for cross-sectional studies comprises 11 items. Study quality is typically graded as follows: 8–11 points = high quality; 5–7 points = moderate quality; 0–4 points = low quality. For retrospective studies, quality was appraised with the Newcastle–Ottawa Scale (NOS) for case control studies (8). The maximum NOS score is 9; studies scoring ≥ 6 are classified as high quality, whereas those < 6 are considered low quality.

### 2.6 Statistical analysis

RevMan 5.4 software was used for meta-analysis. The outcome indicators of the included studies were expressed by mean ± SD. When studies did not report the mean ± SD, we applied the method recommended by the Cochrane Handbook. Using the reported minimum, first quartile, median, third quartile, maximum, and sample size, we estimated the sample mean and SD according to the approaches proposed by Luo et al. (9) and Wan et al. (10).The Q test was used for heterogeneity test: P < 0.1 or I² > 50%, indicating obvious heterogeneity among the results of each study, and the random effect model was used. P > 0.1 or I² < 50% indicated that there was little heterogeneity among the results of each study, and the fixed effect model was used. Stata 18.0 software was used to analyze the publication bias of each outcome indicator, and the publication bias was analyzed in the form of funnel plot. P < 0.05 was considered statistically significant.

## 3 Results

### 3.1 Results of literature search

**Figure 1:**
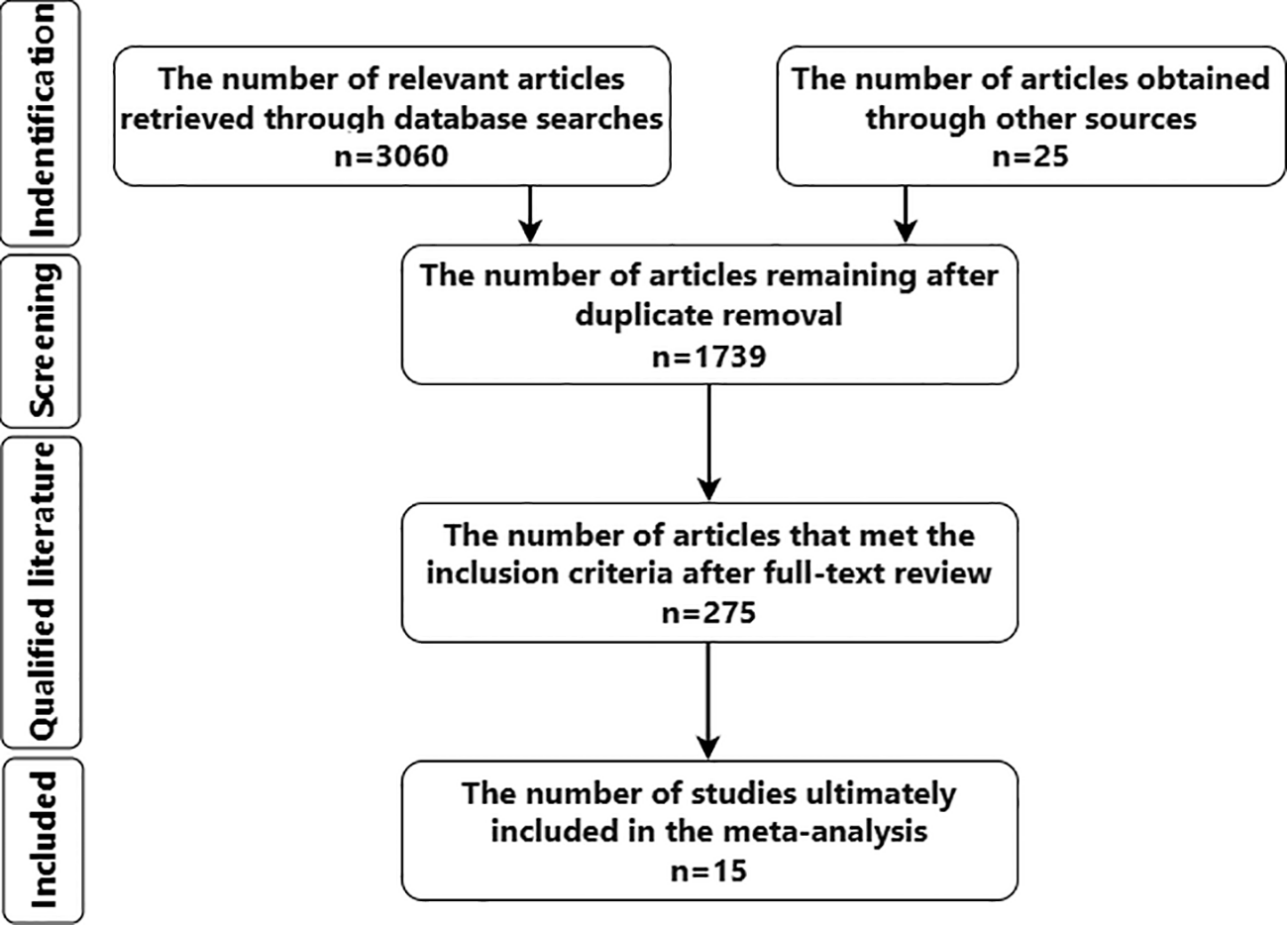
The flow diagram for identifying studies.

A total of 275 relevant studies were retrieved, and 14 studies were finally included (The flow diagram for identifying studies was shown in **Figure 1**). All of them were cohort studies in English, with a total of 8443 patients, including 3467 normal weight patients, 3444 overweight patients, and 1532 obese patients. All 14 studies involved semen quality analysis, and 5 studies involved sex hormone quality analysis 13 studies were cross-sectional, and 1 was retrospective. Nine studies received a quality score of 8, while five scored 7. (Basic characteristics of the included studies was shown in **Table 1**, the detailed individual literature quality assessment scales were provided in **Supplementary Material 1**).

**Table 1:** Basic characteristic of the included studies.

Author, Year	Nation	Age	Study type	Normal weight	Overweight	Obesity	Population	Quality evaluation
Gill, 2025 (13)	Poland	33.19 ± 6.72	Cross-sectional	209	218	116	Volunteers	7(Moderate)
Bandel, 2015 (23)	Sweden	27.90 ± 10.99	Cross-sectional	905	456	115	In the middle	8(High)
L. V. Osadchuk, 2023 (14)	Russia	22.50 ± 3.07	Cross-sectional	147	62	17	Volunteers	7(Moderate)
Leila, 2020 (15)	Iran	34.64 ± 5.87	Cross-sectional	30	56	33	Infertile men	8(High)
Ma, 2020 (17)	China	30.92 ± 4.86	Cross-sectional	103	54	20	Volunteers	8(High)
N.V. Gutorova, 2014 (24)	Russia	37.90 ± 0.24	Cross-sectional	36	44	19	Volunteers	7(Moderate)
Nataliia, 2020 (18)	Ukraine	32.58 ± 6.98	Cross-sectional	63	66	23	Infertility men	7(Moderate)
Oliveira, 2017 (19)	Brazil	37.90 ± 6.60	Cross-sectional	370	856	598	Infertility men	8(High)
Ramaraju, 2017 (20)	India	34.50 ± 4.70	Retrospective cohort	473	611	201	Infertility men	7(Moderate)
Tang, 2015 (21)	China	32.00 ± 5.20	Cross-sectional	334	220	63	Infertility men	8(High)
Ehala-Aleksejev, 2015 (22)	Estonia	32.3 ± 6.70	Cross-sectional	127	95	38	Volunteers	8(High)
Emad, 2016 (12)	Egypt	36.53 ± 4.89	Cross-sectional	81	59	25	Fertile men	7(Moderate)
Márton, 2020 (16)	Hungary	38.10 ± 7.00	Cross-sectional	438	510	221	Infertility men	8(High)
Charlotte, 2013 (11)	France	37.60 ± 6.20	Cross-sectional	151	137	43	Subfertile	8(High)

### 3.2 Publication bias analysis

**Figure 2:**
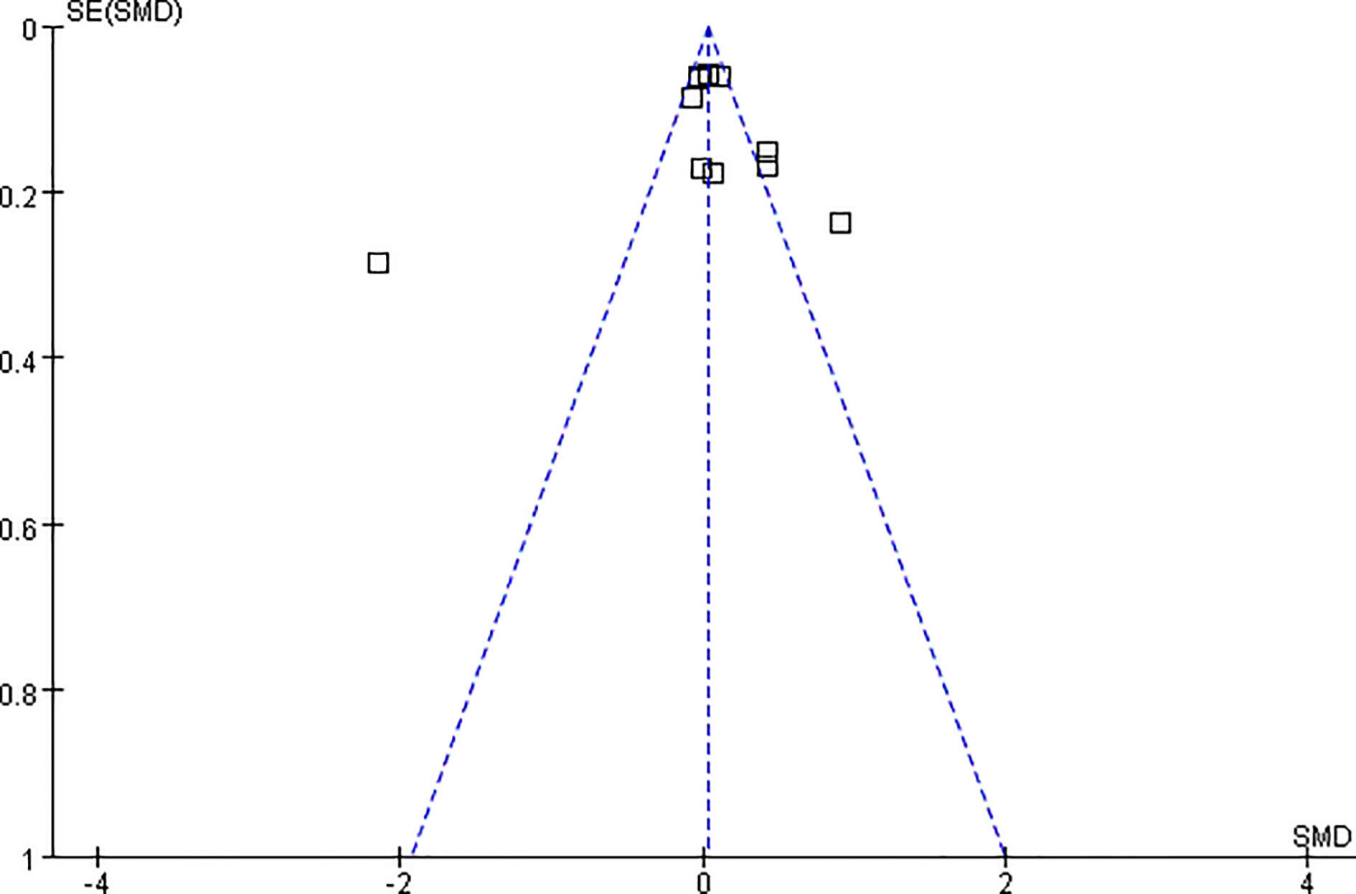
Funnel plot of publication bias.

Funnel plots were visually inspected for symmetry, and Egger’s linear regression was used to test for publication bias. The funnel plot appeared symmetrical (**Figure 2**), and Egger’s test was non-significant (intercept = 3.01, 95% CI: -1.73~7.76; P = 0.192), indicating no evidence of substantial publication bias.

### 3.3 Results of meta-analysis

#### 3.3.1 Meta-analysis of sperm quality

NM (%): A total of 12 studies were involved in the analysis of NM (11–22). The results of heterogeneity test indicated P<0.0001, I^2^ = 89%, there was statistical heterogeneity, so the random effect model was used. The results of meta-analysis showed MD = 0.48 (0.23~0.72), P = 0.0001. Subgroup analysis results showed that PM was statistically significant in normal weight versus obesity (P = 0.004) and overweight versus obesity individuals (P = 0.01).

TM (%): A total of 4 studies were involved in the analysis of TM (11, 13, 19, 20). The results of heterogeneity test indicated P = 0.006, I^2^ = 58%, there was statistical heterogeneity, so the random effect model was used. The results of meta-analysis showed MD = 3.88, 95% CI: 2.46~5.30, P<0.001. Subgroup analysis results showed that PM was statistically significant in normal weight versus overweight (P = 0.01), normal weight versus obesity (P<0.001) and overweight versus obesity individuals (P<0.001).

SC (x10^6^/ml): A total of 13 studies were involved in the analysis of SC (12–24). The results of heterogeneity test indicated P<0.0001, I^2^ = 92%, there was statistical heterogeneity, so the random effect model was used. The results of meta-analysis showed MD = 7.46 (2.72~12.21), P = 0.002. Subgroup analysis results showed that volume was statistically significant in normal weight versus obesity (P = 0.001).

PM (%): A total of 10 studies were involved in the analysis of sperm PM (12–16, 18–21, 23). The results of heterogeneity test indicated P<0.0001, I^2^ = 89%, there was statistical heterogeneity, so the random effect model was used. The results of meta-analysis showed MD = 4.29 (2.48~6.10), P <0.0001. Subgroup analysis results showed that PM was statistically significant in normal weight versus overweight ((P = 0.04), normal weight versus obesity (P = 0.002) and overweight versus obesity individuals (P = 0.002).

Volume (ml): A total of 11 studies were involved in the analysis of semen volume (13–15, 17–24). The results of heterogeneity test indicated P<0.0001, I^2^ = 76%, there was statistical heterogeneity, so the random effect model was used. The results of meta-analysis showed MD = 0.16 (0.06~0.26), P = 0.002. Subgroup analysis results showed that volume was statistically significant in overweight versus obesity individuals (P<0.0001).

SDF (%): A total of 5 studies were involved in the analysis of SDF (11, 12, 14, 19, 23). The results of heterogeneity test indicated P<0.0001, I^2^ = 96%, there was statistical heterogeneity, so the random effect model was used. The results of meta-analysis showed MD = -1.48 (-3.09~0.12), P = 0.07. There was no statistically significant after subgroup analysis.

**Figure 3:**
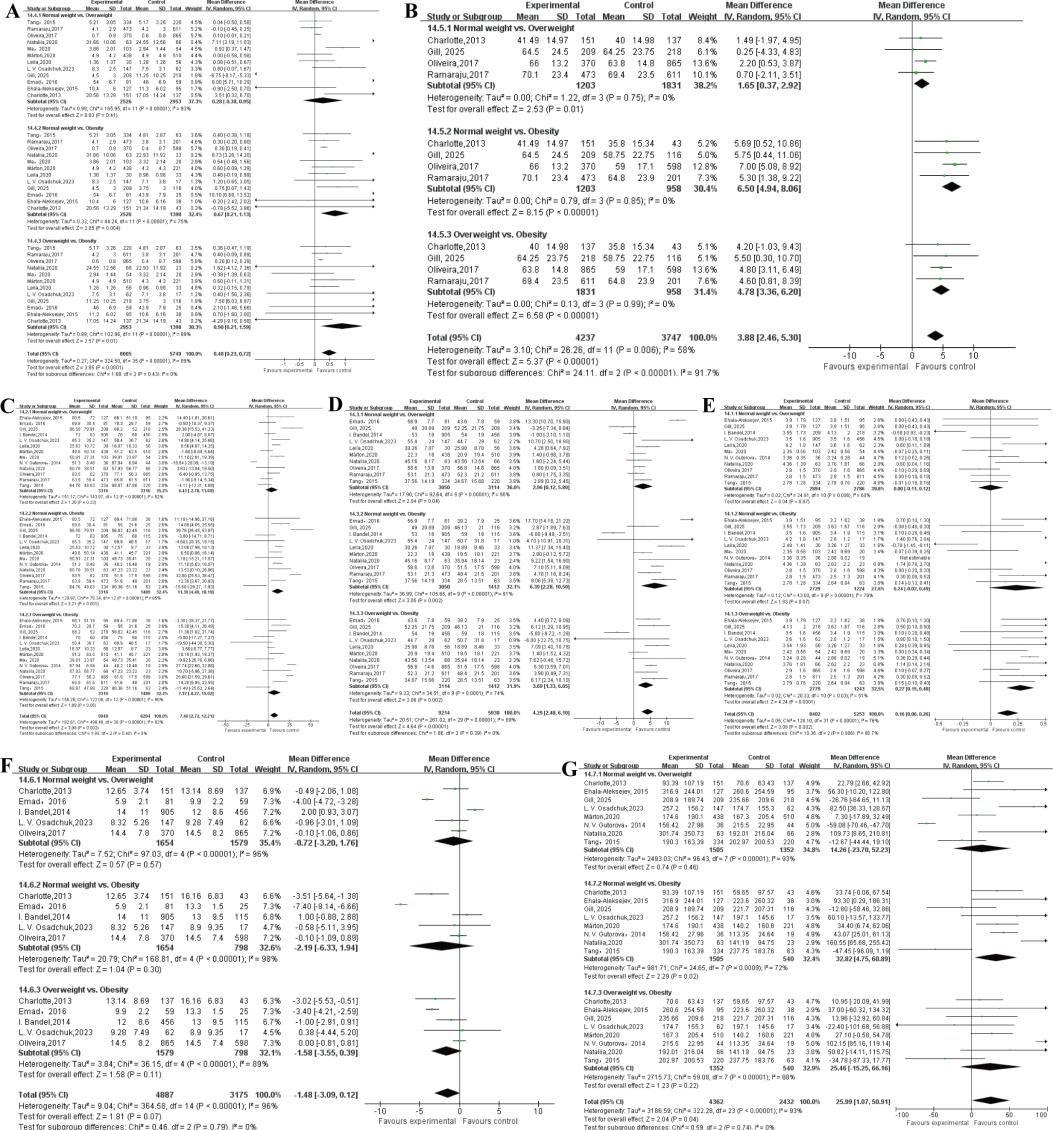
The results of the meta-analysis of sperm quality. **(A)** normal morphology. **(B)** total motility. **(C)** sperm concentration. **(D)** progressive motility (%). **(E)** sperm DNA fragmentation. **(F)** total sperm count.

TSC (10^6^): A total of 7 studies were involved in the analysis of TSC (11, 13, 14, 16, 18, 21, 22, 24). The results of heterogeneity test indicated P<0.0001, I^2^ = 93%, there was statistical heterogeneity, so the random effect model was used. The results of meta-analysis showed MD = 25.99 (1.07~50.91), P = 0.04. There was no statistically significant after subgroup analysis. Subgroup analysis results showed that PM was statistically significant in normal weight versus obesity (P = 0.02). The results of the meta-analysis of sperm quality were presented in **Table 2**; **Figure 3**.

**Table 2:** The results of the meta-analysis of sperm quality.

Variable	Normal weight vs. overweight		Normal weight vs. obesity		Overweight vs. obesity		Overall	
	P	MD (95% CI)	P	MD (95% CI)	P	MD (95% CI)	P	MD (95% CI)
NM (%)	0.41	0.28 (-0.38~0.95)	0.004	0.67 (0.21~1.13)	0.01	0.90 (0.21~1.59)	<0.0001	0.48 (0.23~0.72)
TM (%)	0.01	1.65(0.37~2.92)	<0.0001	6.50(4.94~8.06)	<0.0001	4.78(3.36~6.20)	<0.0001	3.88(2.46~5.30)
SC (x10^6^/ml)	0.23	4.41(-2.78~11.60)	0.001	11.30(4.40~18.19)	0.06	7.37(-0.27~15.02)	0.002	7.46(2.72~12.21)
PM (%)	0.04	2.96 (0.12~5.80)	0.002	6.39(2.28~10.50)	0.002	3.69(1.33~6.05)	<0.0001	4.29(2.48~6.10)
Volume(ml)	0.97	0.00 (-0.11~0.12)	0.07	0.24(-0.02~0.49)	<0.0001	0.27 (0.15~0.40)	0.002	0.16 (0.06~0.26)
SDF (%)	0.57	-0.72(-3.20~1.76)	0.30	-2.19(-6.33~1.94)	0.11	-1.58(-3.55~0.39)	0.07	-1.48(-3.09~0.12)
TSC (10^6^)	0.46	14.26 (-23.70~53.23)	0.02	32.82(4.75~60.89)	0.22	25.46(-15.25~66.16)	0.04	25.99(1.07~50.91)

NM, normal morphology; TM, total motility; SC, sperm concentration; PM, progressive motility (%); SDF, sperm DNA fragmentation; TSC, total sperm count.

#### 3.3.2 Meta-analysis stratified by study population

Of the 14 included studies, six recruited volunteer donors and six enrolled infertile men. Stratified analyses were therefore performed to compare semen quality between normal-weight and obese men within each population. The pooled results remained consistent with the overall meta-analysis: normal-weight men exhibited significantly higher NM (P = 0.001), SC (P = 0.004), PM (P = 0.006),and TSC (P<0.0001) than obese male (the results of the meta-analysis of sperm quality stratified by study population were presented in **Table 3**; **Supplementary Material 2**). Crucially, the same negative pattern was observed in both volunteers and infertile men, underscoring that obesity itself exerts an independent and deleterious effect on semen quality.

**Table 3:** Meta-analysis of semen quality differences between normal weight and obese male, stratified by study population (volunteers vs. infertile men).

Variable	Volunteers				Infertile men				Total			
	Heterogeneity		Test for overall effect		Heterogeneity		Test for overall effect		Heterogeneity		Test for overall effect	
	P	I^2^	P	95%CI	P	I^2^	P	95%CI	P	I^2^	P	95%CI
NM (%)	0.18	39	0.01	0.99(0.20~1.79)	0.06	54	0.02	0.44(0.07~0.80)	0.02	54	0.001	0.58(0.23~0.92)
SC (x10^6^/ml)	0.08	56	<0.0001	38.43(22.31~54.55)	0.0002	88	0.05	23.22(-0.09~46.53)	0.0004	76	<0.0001	33.51(20.25~46.77)
PM (%)	0.004	77	0.86	-0.43(-5.14~4.28)	0.009	67	<0.0001	7.01(4.51~9.50)	<0.0001	87	0.006	4.48(1.27~7.68)
Volume(ml)	0.002	74	0.92	-0.01(-0.21~0.19)	0.0002	82	0.37	0.16(-0.19~0.51)	0.0001	77	0.54	0.05(-0.12~0.22)
TSC (10^6^)	0.08	56	<0.0001	38.43(22.31~54.55)	0.0002	88	0.05	23.22(-0.09~46.53)	0.0004	76	<0.0001	33.51(20.25~46.77)

NM, normal morphology; SC, sperm concentration; PM, progressive motility (%); SC, total sperm count.

#### 3.3.3 Meta-analysis of sex hormones

TT (nmol/l): A total of 4 studies were involved in the analysis of TT (14, 15, 17, 24). The results of heterogeneity test indicated P<0.0001, I^2^ = 98%, there was statistical heterogeneity, so the random effect model was used. The results of meta-analysis showed MD = 1.78 (1.60~1.96), P<0.0001. Subgroup analysis results showed that volume was statistically significant in normal weight versus overweight (<0.0001), normal weight versus obesity (<0.0001) and overweight versus obesity individuals (P<0.0001).

E2 (nmol/l): A total of 4 studies were involved in the analysis of E2 (14, 15, 17, 24). The results of heterogeneity test indicated P<0.0001, I^2^ = 73%, there was statistical heterogeneity, so the random effect model was used. The results of meta-analysis showed MD = 0.01 (-0.01~0.02), P = 0.33. There was no statistically significant after subgroup analysis.

FSH (IU/l): A total of 4 studies were involved in the analysis of FSH (14, 15, 17, 24). The results of heterogeneity test indicated P<0.0001, I^2^ = 93%, there was statistical heterogeneity, so the random effect model was used. The results of meta-analysis showed MD = -0.68 (-1.19~-0.17), P = 0.009. There was no statistically significant after subgroup analysis.

**Figure 4:**
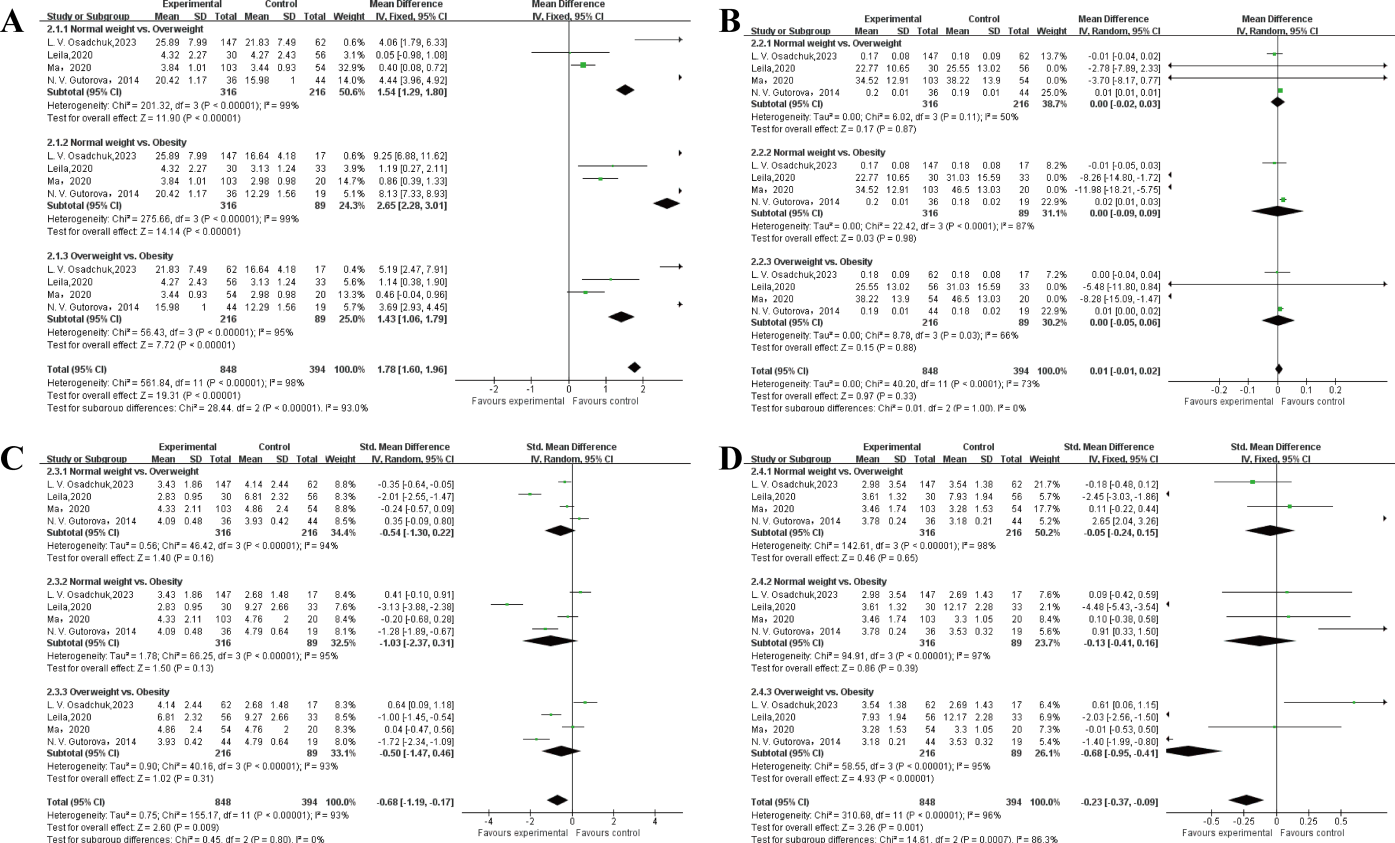
The results of the meta-analysis of sex hormone. **(A)** total testosterone. **(B)** estradiol. **(C)** follicle stimulating hormone. **(D)** luteinizing hormone.

LH (IU/l): A total of 4 studies were involved in the analysis of LH (14, 15, 17, 24). The results of heterogeneity test indicated P<0.0001, I^2^ = 96%, there was statistical heterogeneity, so the random effect model was used. The results of meta-analysis showed MD = -0.23 (-0.37~-0.09), P<0.0001. Subgroup analysis results showed that volume was statistically significant in overweight versus obesity individuals (P = 0.001). The results of the meta-analysis of sperm quality were presented in **Table 4** and **Figure 4**.

**Table 4:** The results of the meta-analysis of sex hormones.

Variable	Normal weight vs. overweight		Normal weight vs. obesity		Overweight vs. obesity		Overall	
	P	MD (95% CI)	P	MD (95% CI)	P	MD (95% CI)	P	MD (95% CI)
TT (nmol/L)	<0.0001	1.54 (1.29~1.80)	<0.0001	2.65 (2.28~3.01)	<0.0001	1.43 (1.06~1.79)	<0.0001	1.78 (1.60~1.96)
E2 (pmol/L)	0.87	0.00(-0.02~0.03)	0.98	0.00(-0.09~0.09)	0.88	0.00(-0.05~0.06)	0.33	0.01(-0.01~0.02)
FSH (IU/L)	0.16	-0.54(-1.30~0.22)	0.13	-1.03(-2.37~0.31)	0.31	-0.50(-1.47~0.46)	0.009	-0.68(-1.19~-0.17)
LH (IU/L)	0.65	-0.05(-0.24~0.15)	0.39	-0.13(-0.41~0.16)	<0.0001	-0.68(-0.95~-0.41)	0.001	-0.23(-0.37~-0.09)

TT, total testosterone; E2, estradiol; FSH, follicle stimulating hormone; LH, luteinizing hormone.

## 4 Discussion

This meta-analysis systematically evaluated the association between BMI and semen quality as well as reproductive hormone levels in male. The results showed that, compared with normal weight, overweight and obese men exhibited significantly lower NM, TM, SC, PM, semen volume and TSC. Regarding reproductive hormones, TT, LH and FSH also differed significantly across BMI categories. These findings are consistent with most previous studies and further confirm that BMI is an important and modifiable key factor affecting male fertility (25, 26).

However, we observed substantial heterogeneity when pooling semen parameters, with I² reaching 96% for DFS. Although the studies included in this meta-analysis were all rated as moderate-to-high quality, ensuring the reliability of study design and basic methodology, the pooled results still exhibited substantial statistical heterogeneity. This suggests that current quality-assessment tools, such as AHRQ and NOS, primarily focus on internal validity and reporting adequacy, but cannot fully capture other important sources of between-study variability. The included studies enrolled both sub-fertile men attending fertility clinics and healthy volunteers drawn from sperm banks or the general public. These two groups differ systematically in baseline fertility, reasons for taking part, co-morbidities and lifestyle habits, all of which can independently influence semen characteristics and thus introduce heterogeneity. To investigate this issue, we stratified the analyses by participant source. As shown in **Table 3**, separating studies into volunteer and infertile subgroups markedly reduced heterogeneity for most semen indices, including SC, PM and NM. This finding indicates that participant origin is a major driver of heterogeneity in our meta-analysis. Importantly, the adverse effect of obesity on semen quality remained evident and consistent in both strata, reinforcing the conclusion that obesity exerts an independent detrimental effect on spermatogenesis.

Apart from the origin of participants, other factors may also contribute to heterogeneity. Geographic and ethnic differences, for instance, can influence the relationship between BMI and reproductive health through distinct genetic backgrounds, dietary habits, and environmental exposures (27–29). In addition, although all included studies stated that they followed the WHO manual for semen analysis, subtle variations in laboratory protocols, technician training, and quality control procedures may introduce additional variability (30). It is worth noting that this study did not find a significant association between SDF and BMI. This result aligns with some previous reports but contradicts others (31). Such inconsistency is most likely due to the diversity of SDF assays, including SCSA, TUNEL and SCD, and to their varying degrees of standardization, both of which inevitably introduce heterogeneity among studies.

Importantly, our analyses revealed that obesity exerts a disproportionately greater adverse effect on semen quality than overweight, while the difference between normal weight and overweight individuals remains relatively modest. Although a few comparisons did not reach the conventional threshold of P < 0.05, the magnitude of decline in semen parameters was consistently larger between normal-weight and obese men, reinforcing the notion of a threshold effect rather than a purely linear relationship between BMI and spermatogenesis. These results were similar with the dose–response analysis reported by Guo et al. (32), who found that every 5-unit rise in BMI was associated with a 2.4%, 1.3% and 2.0% reduction in total sperm count, sperm concentration and semen volume, respectively (expressed as SMD).

From a mechanistic perspective, obesity impairs male fertility through multiple pathways (33, 34). Adipose tissue, especially visceral fat, releases inflammatory cytokines and free fatty acids that trigger chronic local inflammation and oxidative stress within the testis. These factors disrupt the blood-testis barrier and inhibit testosterone synthesis by Leydig cells (35, 36). Reduced testosterone undermines the stability of the spermatogenic microenvironment, blocks spermatogenesis and impedes sperm maturation, providing a pathophysiologic basis for the observed declines in SC, TM and NM. Meanwhile, dysregulation of feedback control along the hypothalamic pituitary gonadal axis may partly explain the alterations in FSH levels (26, 37).

Although the inflammation and oxidative stress are of significant concern, an array of studies suggests that dietary adjustments and physical exercise can effectively mitigate and potentially reverse obesity-related comorbidities. Andersen et al. (38) found that an 8-week diet-induced weight loss program improved SC and TSC in asthenozoospermia patients. Mir et al. (39) reported that in a weight-loss program guided by nutrition and exercise, SDF was significantly reduced. Sharma et al. (40) found that both low-energy and simple dietary interventions similarly improved TM in obese men, whereas more intensive dietary regimens conferred even greater benefits for asthenozoospermia patients. Xu et al. (41) demonstrated that both moderate-intensity continuous training and high intensity interval training protect testicular tissue in high fat diet mice from oxidative stress, apoptosis, and m6A methylation damage, resulting in improved testicular morphology and function.

Limitations of this study include the following. Although the total sample size exceeded eight thousand participants, the number of studies available for certain indicators such as some hormones remained small. Publication bias was also present. We were unable to fully explore all potential sources of heterogeneity, including specific dietary patterns, exercise frequency and detailed laboratory protocols. However, by conducting subgroup analyses based on the source of participants, we effectively identified and partially explained the main heterogeneity, thereby strengthening the reliability of our core findings.

## 5 Conclusion

The findings of this study demonstrate that elevated BMI is significantly associated with decreased semen quality and altered sex hormone levels in men. When interpreting the relevant clinical evidence, the heterogeneity possibly introduced by the source of study participants should be fully taken into account. Given that BMI exerts a consistently negative effect on semen parameters across different populations, BMI assessment should be regarded as a routine and essential component of male fertility evaluation in clinical practice. Future investigations should employ more refined designs, recruit more homogeneous cohorts, and incorporate comprehensive covariates to further elucidate the specific pathways through which BMI affects male reproductive health and to provide higher-level evidence supporting fertility improvements in obese men via lifestyle interventions.

## Data Availability

The original contributions presented in the study are included in the article/**Supplementary Material**. Further inquiries can be directed to the corresponding author.
