# Supplementary material for: BMI is associated with sperm quality and sex hormones in men: a meta-analysis
Source: Front Endocrinol (Lausanne). 2025 Dec 5;16:1714019. doi: 10.3389/fendo.2025.1714019 (PMC12714629; doi:10.3389/fendo.2025.1714019)
Supplement: Supplementary file 2 [file DataSheet2.docx]

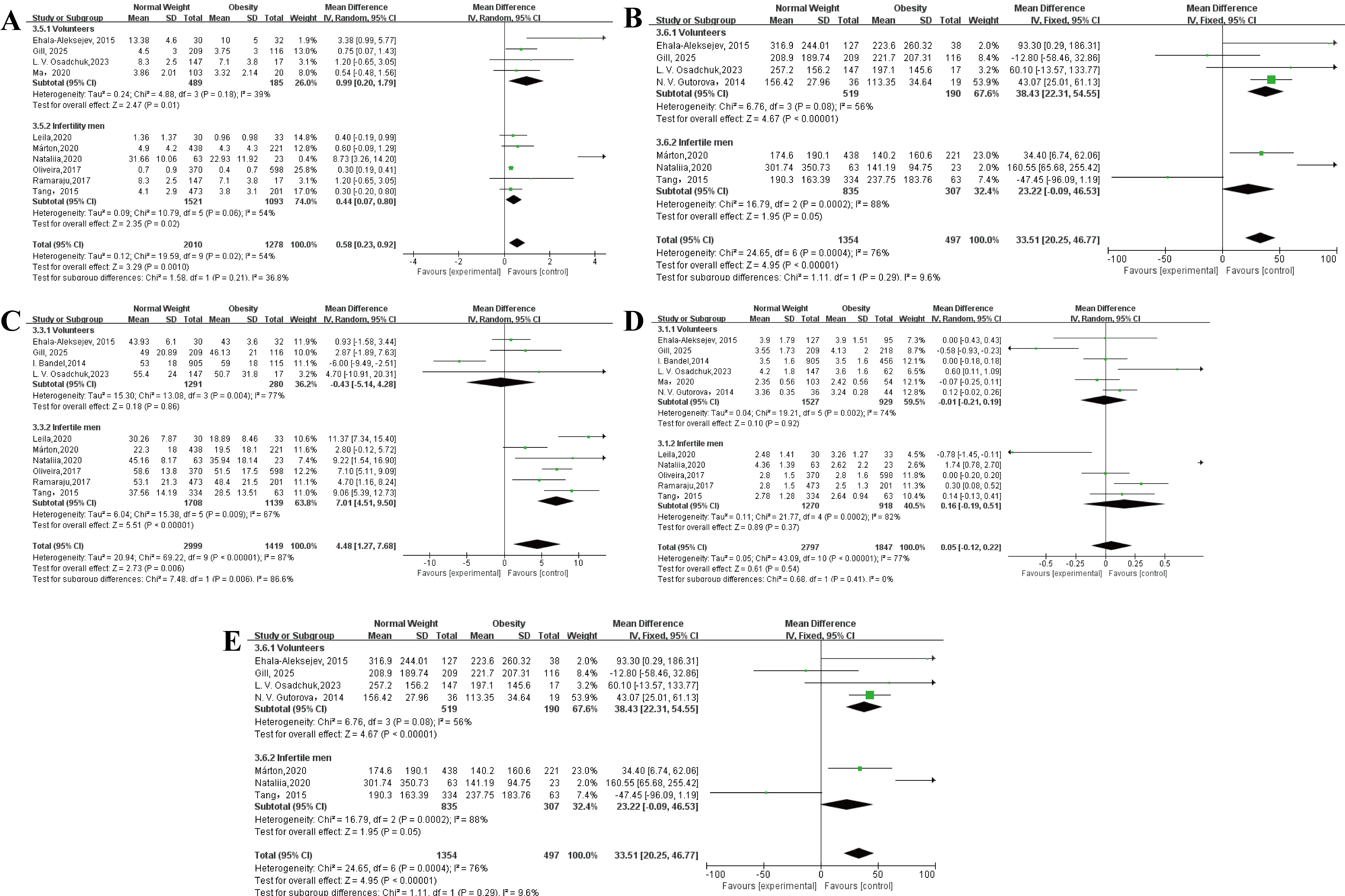


S2：The results of the meta-analysis of sperm quality stratified by study population

A: normal morphology, B: sperm concentration, C: progressive motility (%), D: volume，E:total sperm count
